# Supplementary material for: Bile-mediated ToxS homodimerization provides a model for ToxRS periplasmic interactions
Source: bioRxiv. 2026 Mar 28:2026.01.06.697910. Preprint. [Version 2] doi: 10.64898/2026.01.06.697910 (PMC13042144; doi:10.64898/2026.01.06.697910)
Supplement: Supplement 1 [file media-1.pdf]

Bile-mediated ToxS homodimerization provides a model for ToxRS periplasmic interactions

Minje Kim[1], Deepak Balasubramanian[2], F. Jon Kull[1], Salvador Almagro-Moreno[2,3]*, Charles R. Midgett[1]*

[1]Department of Chemistry, Dartmouth College, Hanover, NH, USA

[2]Department of Host-Microbe Interactions, St. Jude Children's Research Hospital, Memphis, TN, USA

[3]St. Jude Children's Research Hospital Graduate School of Biomedical Sciences, St. Jude Children's Research Hospital, Memphis, TN, USA

*For correspondence: Charles R. Midgett; charles.r.midgett@dartmouth.edu (CRM), Salvador Almagro-Moreno; samoreno@stjude.org (SAM)

Running title: Bile mediates ToxS periplasmic domain homodimerization

Supplemental Information

Table S1. Data collection and refinement statistics for ToxSp.

	**Vp-ToxSp** (PDB ID: 8U2F)	**GCH bound Vp-ToxSp** (PDB ID: 9ZJO)
**Wavelength (Å)**	0.920112	0.920105
**Resolution range (Å)**	26.92 - 2.004 (2.076 - 2.004)	66.55 - 1.961 (2.01 - 1.96)
**Space group**	P 21 21 21	P 21 21 21
**Unit cell (Å, º)**	53.85 58.33 89.68 90 90 90	69.828 92.503 95.805 90 90 90
**Total reflections**	172599 (17411)	274081 (18185)
**Unique reflections**	19566 (1912)	44411 (2966)
**Multiplicity**	8.8 (9.1)	6.2 (6.1)
**Completeness (%)**	99.84 (99.69)	97.63 (98.15)
**Mean I/sigma(I)**	10.99 (0.94)	6.71 (1.06)
**Wilson B-factor (Å^2)**	43.25	27.88
**R-merge**	0.1052 (1.984)	0.207 (2.569)
**R-meas**	0.112 (2.103)	0.2264 (2.809)
**R-pim**	0.03781 (0.6912)	0.09038 (1.124)
**CC1/2**	0.998 (0.526)	0.995 (0.439)
**CC***	1 (0.83)	0.999 (0.781)
**Reflections used in refinement**	19551 (1912)	44103 (2911)
**Reflections used for R-free**	1958 (191)	2160 (147)
**R-work**	0.2252 (0.3039)	0.2013 (0.2717)
**R-free**	0.2711 (0.3369)	0.2343 (0.3098)
**CC(work)**	0.938 (0.769)	0.959 (0.759)
**CC(free)**	0.897 (0.748)	0.974 (0.741)
**Number of non-hydrogen atoms**	2334	5591
**macromolecules**	2243	4772
**ligands**	0	271
**solvent**	91	548
**Protein residues**	281	596
**RMS(bonds) (Å)**	0.010	0.009
**RMS(angles) (°)**	1.38	1.07
**Ramachandran favored (%)**	100.00	98.13
**Ramachandran allowed (%)**	0.00	1.87
**Ramachandran outliers (%)**	0.00	0
**Rotamer outliers (%)**	0.38	0.71
**Clashscore**	13.77	5.31
**Average B-factor (Å^2)**	50.02	33.91
**macromolecules**	49.89	31.92
**ligands**		58.99
**solvent**	53.09	38.86

Table S2. Sequence alignment and chemical properties of ToxSp binding pocket from 280 Vibrionaceae species.

ToxSp Seq. Alignment Position	Vp-ToxSp Seq. Position	Vp-ToxSp AA	% Identity	% Hydrophobic	% Hydrophilic	% Charged	% Aromatic	% Pro/Gly
43	41	Met (M)	53.93	66.43	32.86	0.71	0	0
45	43	Thr (T)	57.86	13.21	82.50	0.71	1.43	1.79
47	45	Ile (I)	74.64	97.86	0.36	0.36	1.43	0
49	47	Asp (D)	26.43	9.29	25.36	51.07	2.86	8.93
51	49	Leu (L)	21.79	36.79	21.07	33.93	0.71	5.00
57	50	Pro (P)	7.86	17.14	43.57	13.93	9.64	8.93
64	54	Val (V)	33.93	70.00	6.79	1.43	0.71	0
70	57	Leu (L)	92.86	99.64	0.36	0	0	0
73	60	Val (V)	68.93	93.57	5.71	0	0	0.71
75	62	Val (V)	37.86	75.71	19.29	3.57	0.71	0.71
91	76	Arg (R)	90.36	0.71	0	99.29	0	0
95	80	Ile (I)	22.5	99.29	0	0	0.71	0
97	82	Leu (L)	86.79	95.00	0	0	4.64	0
116	93	Ile (I)	71.07	98.57	0	0	1.43	0
158	134	Ile (I)	80	100.00	0	0	0	0
161	137	Ile (I)	28.93	77.50	0	1.43	20.71	0
162	138	Phe (F)	67.5	22.50	0	0	77.50	0
165	141	Asp (D)	73.57	0.71	13.93	80.36	0	4.64

To generate the percent identity, the frequency of each amino acid in the alignment was determined using UGENE [1]. Applying a STATA (StataCorp) script, the frequency was used to calculate the percentage of each amino acid at each position. To obtain the percentages of hydrophobic, hydrophilic, charged, aromatic, and pro/gly the percentages of the selected amino acids for each category added together.

1. Okonechnikov K, Golosova O, Fursov M, Varlamov A, Vaskin Y, Efremov I, et al. Unipro UGENE: a unified bioinformatics toolkit. Bioinformatics. 2012;28: 1166–1167. doi:10.1093/BIOINFORMATICS/BTS091

Table S3. DALI search result showing Vp-ToxSp structural similarity with chaperone proteins.

No	PDB ID-Chain	Z	rmsd	lali	nres	%id PDB	Description
1	5kew-B	10.6	2.6	109	132	9 PDB	MOLECULE VTRA PROTEIN;
2	4nyq-A	9.3	2.7	98	153	9 PDB	MOLECULE MILK PROTEIN;
3	4u3q-B	9.2	2.1	86	99	17 PDB	MOLECULE 17 KDA LIPOPROTEIN;
4	2kts-A	8.8	3.1	97	117	7 PDB	MOLECULE HEAT SHOCK PROTEIN HSLJ;
5	2la7-A	8.1	2.8	103	145	13 PDB	MOLECULE UNCHARACTERIZED PROTEIN;
6	3lhn-A	8	2.7	91	108	8 PDB	MOLECULE LIPOPROTEIN;
7	4n7c-A	7.7	2.8	100	174	9 PDB	MOLECULE BLA G 4 ALLERGEN VARIANT 1;
8	2erv-A	7.7	3.1	98	150	3 PDB	MOLECULE HYPOTHETICAL PROTEIN PAER03002360;
9	4kh8-A	7.7	2.4	90	311	10 PDB	MOLECULE HYPOTHETICAL PROTEIN;
10	4rlc-A	7.7	3.2	102	135	7 PDB	MOLECULE OUTER MEMBRANE PORIN F;
11	4iab-A	7.5	4.2	95	142	13 PDB	MOLECULE HYPOTHETICAL PROTEIN;
12	2jw1-A	7.5	3.1	93	115	13 PDB	MOLECULE LIPOPROTEIN MXIM;
13	2mhd-A	7.5	3	90	110	11 PDB	MOLECULE UNCHARACTERIZED PROTEIN;
14	8ehd-A	7.4	3.4	82	101	13 PDB	MOLECULE POTEMPIN E (POTE);
15	7b2a-A	7.3	2.5	92	162	7 PDB	MOLECULE CIRPA5;
16	1qft-A	7.3	2.7	95	175	7 PDB	MOLECULE PROTEIN (FEMALE-SPECIFIC HISTAMINE BINDING
17	4lqz-A	7.2	2.6	83	131	8 PDB	MOLECULE UNCHARACTERIZED PROTEIN;
18	2z4h-B	7.1	2.8	82	194	13 PDB	MOLECULE COPPER HOMEOSTASIS PROTEIN CUTF;
19	5hcc-C	7	2.5	90	148	6 PDB	MOLECULE COMPLEMENT C5;
20	2m4l-A	6.9	3.4	82	99	6 PDB	MOLECULE PROTEIN BT_0846;
21	1i4u-A	6.9	2.6	90	181	10 PDB	MOLECULE CRUSTACYANIN;
22	3rby-A	6.9	3.4	95	245	8 PDB	MOLECULE UNCHARACTERIZED PROTEIN YLR301W;
23	4tq2-A	6.9	2.8	91	177	12 PDB	MOLECULE PUTATIVE PHYCOERYTHRIN LYASE;
24	6x1k-A	6.9	3.4	97	124	7 PDB	MOLECULE DE NOVO DESIGNED TRANSMEMBRANE BETA-BARREL TMB2.3
25	1jmx-A	6.8	3.5	96	493	7 PDB	MOLECULE AMINE DEHYDROGENASE;

Table S6. Sequence and primers for ToxS constructs.

Protein	Vector	Amino acid sequence
Vp-ToxSp	pTYB21	MSDLKVEQVLTSNEWQSTMVTVITDNLPDDTVGPLRRVNVESNVKYLPNGDYIRVSNIKLFAQGSTAESTINISEKGRWEVSDNYLLVSPSEFKDISSSQSKDFSEAQLRLITQIFKLDAEQSRRIDVVNEKTLLLTSLNHGSTVLFRN

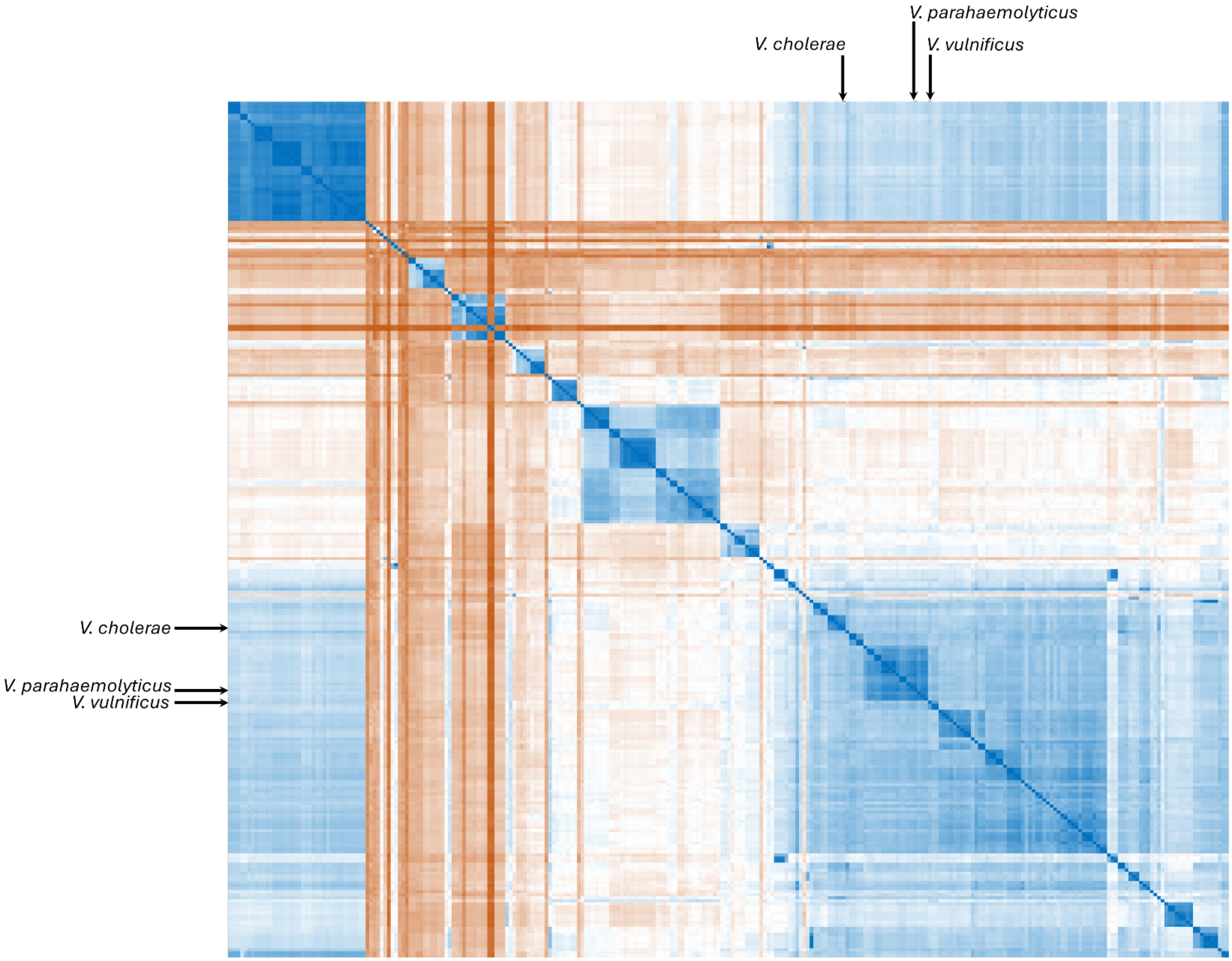

Figure S1. Pairwise percent identity matrix for ToxSp sequence across 280 sequences from Vibrionaceae family. Each pixel shows the pairwise sequence percent identity generated from multiple sequence alignment between 280 Vibrionaceae ToxSp sequences. The diagonal (dark blue) corresponds to self-comparison (100% identity). The rows and columns represent same order of sequences matched pairwise to create a symmetric matrix. The order of the 280 sequences is the same as that of ToxRp matrix for direct comparison. The color indicates percent identity (blue, high identity; white, intermediate identity; orange, low identity). A higher proportion of blue and white pixels in ToxSp matrix compared to the ToxRp matrix shows that ToxSp sequences are generally more similar to one another than ToxRp.

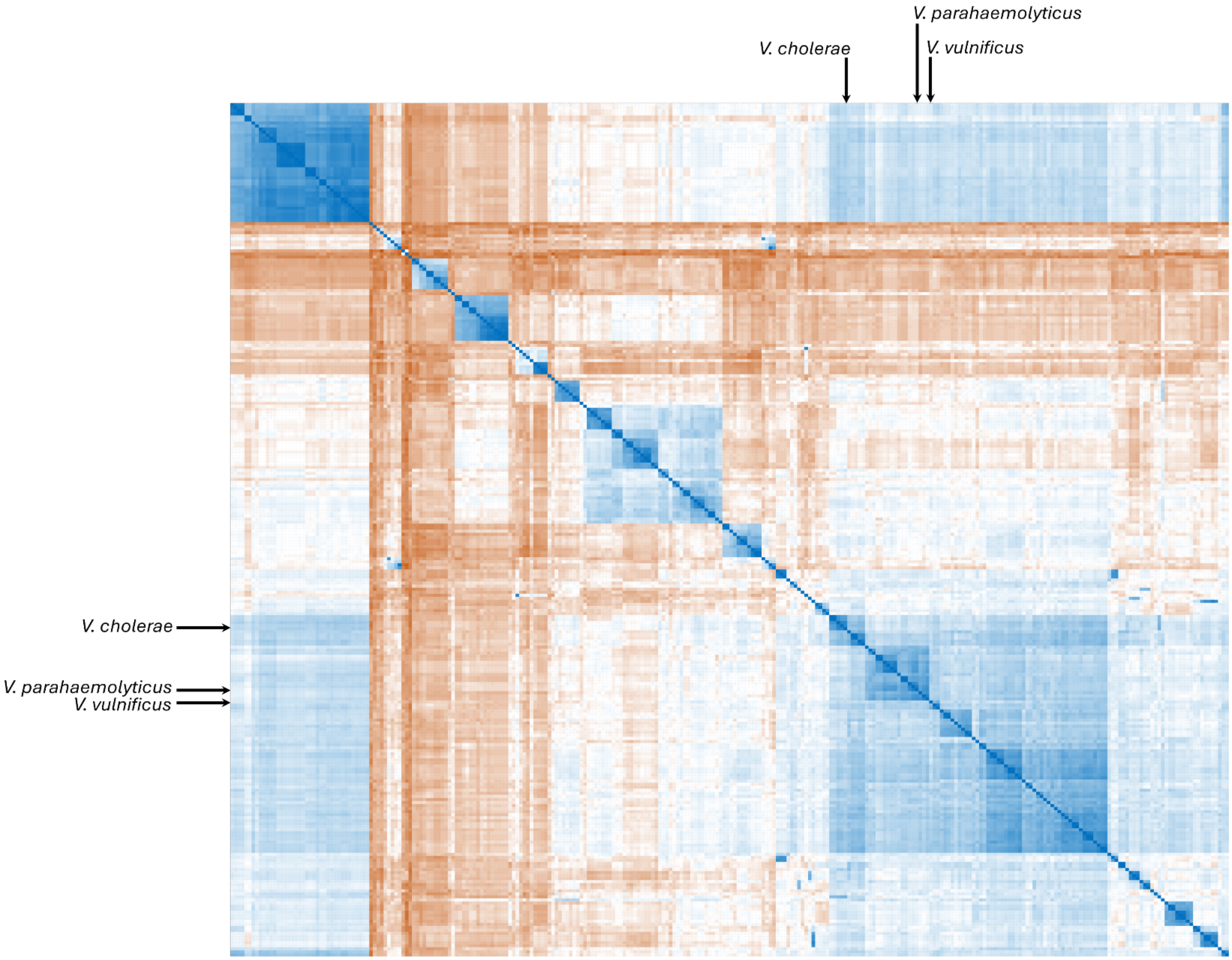

Figure S2. Pairwise percent identity matrix for ToxRp sequence across 280 sequences from Vibrionaceae family in the same order as ToxSp matrix. Each pixel shows the pairwise sequence percent identity generated from multiple sequence alignment between 280 Vibrionaceae ToxRp sequences. The diagonal (dark blue) corresponds to self-comparison (100% identity). The rows and columns represent same ordered list of sequences matched pairwise to create a symmetric matrix. The order of the 280 sequences is the same as that of ToxSp matrix for direct comparison. The color indicates percent identity (blue, high identity; white, intermediate identity; orange, low identity). A higher proportion of orange pixels and fewer blue pixels in the ToxRp matrix compared to ToxSp indicates that ToxRp is less conserved than ToxSp.

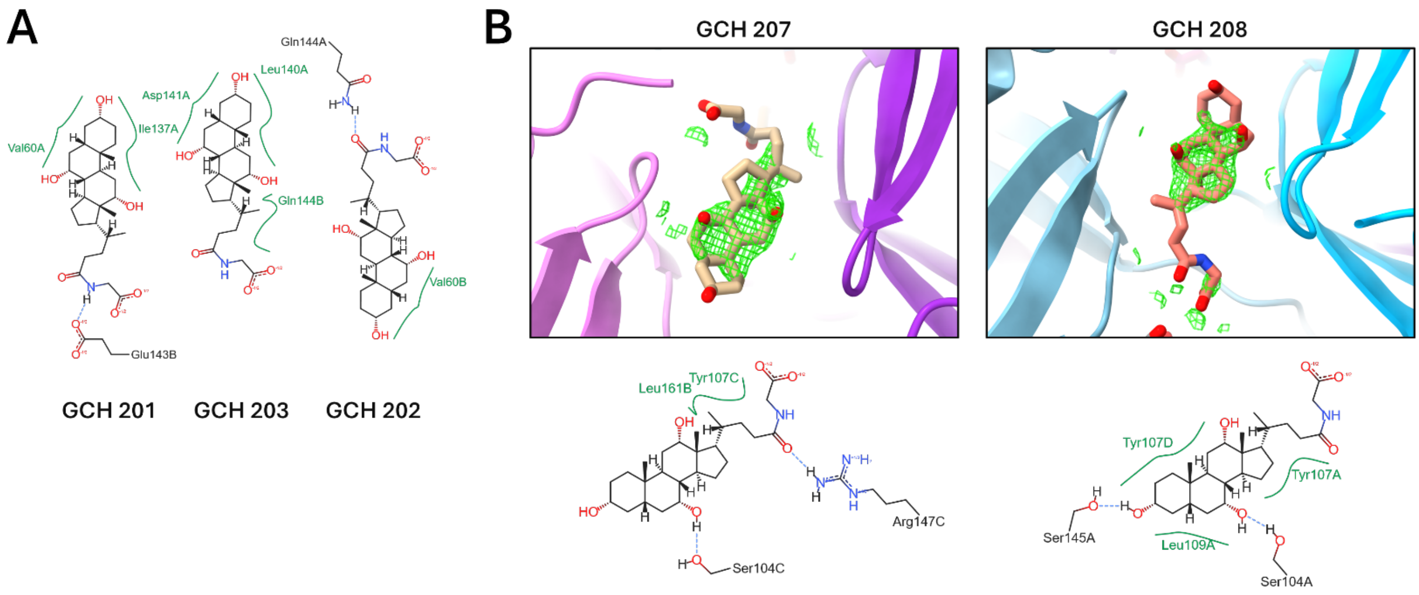

Figure S3. Glycocholate molecules observed at the binding pocket and at the crystal packing sites. (**A**) 2D interaction diagram of the three glycocholate molecules bound between the two chains of the strand-swapped dimer (GCH 201-203) with nearby interacting residues shown. (**B**) Two additional glycocholate molecules observed at the crystal-packing interfaces (GCH 207-208), which are the peripherally bound ligands between the two dimer chains in the crystallographic lattice. 2D interaction diagram of GCH 207-208 are shown alongside the composite omit electron density (green mesh; contoured at 1 σ). Density is the strongest at the ring group and weaker at the glycine-conjugated tail. These ligands are not interpreted as physiologically relevant binding events.
